# Bis(4-methyl-3,5-diphenyl-1*H*-pyrazole-κ*N* ^2^)silver(I) nitrate

**DOI:** 10.1107/S1600536811017776

**Published:** 2011-05-20

**Authors:** Moayad Hossaini Sadr, Behzad Soltani, James T. Engle, Christopher J. Ziegler, M. Kabirzadeh

**Affiliations:** a Department of Chemistry, Azarbaijan University of Tarbiat Moallem, Tabriz, Iran; b Department of Chemistry, University of Akron, Akron, OH, USA

## Abstract

In the title complex, [Ag(C_16_H_14_N_2_)_2_]NO_3_, the geometry around the Ag^I^ ion is T-shaped with two short Ag—N bonds to the pyrazole ligand and one long Ag—O bond to the nitrate anion. The crystal structure is stabilized by inter­molecular N—H⋯O, C—H⋯O and C—H⋯π inter­actions.

## Related literature

For standard bond lengths, see: Allen *et al.* (1987 ▶). For background to pyrazolates and their complexes, see, for example; Rasika Dias *et al.* (2007 ▶); Hossaini Sadr *et al.* (2004 ▶, 2006 ▶, 2008*a* ▶,*b* ▶).
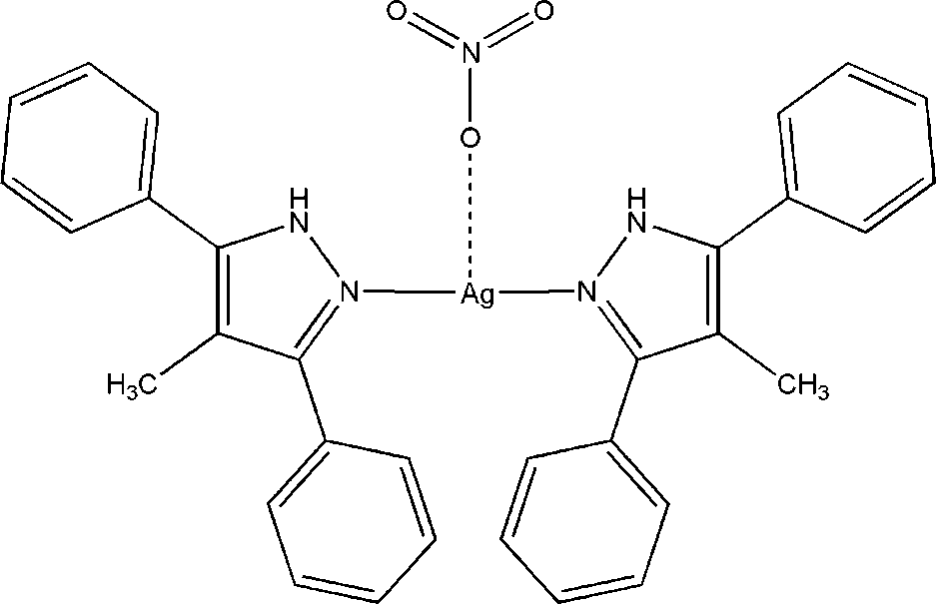


## Experimental

### 

#### Crystal data

[Ag(C_16_H_14_N_2_)_2_]NO_3_
*M* *_r_* = 638.46
Triclinic, 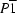

*a* = 10.5529 (12) Å
*b* = 10.8791 (13) Å
*c* = 12.8396 (15) Å
α = 80.454 (2)°
β = 68.806 (2)°
γ = 82.398 (2)°
*V* = 1351.1 (3) Å^3^
*Z* = 2
Mo *K*α radiation
μ = 0.79 mm^−1^
*T* = 100 K
0.45 × 0.25 × 0.08 mm

#### Data collection

Bruker APEXII CCD area-detector diffractometer
Absorption correction: multi-scan (*SADABS*; Bruker, 2005 ▶) *T*_min_ = 0.717, *T*_max_ = 0.940
8897 measured reflections
4588 independent reflections
4127 reflections with *I* > 2σ(*I*)
*R*_int_ = 0.022

#### Refinement

*R*[*F* ^2^ > 2σ(*F* ^2^)] = 0.029
*wR*(*F* ^2^) = 0.084
*S* = 1.15
4588 reflections
372 parameters
H-atom parameters constrained
Δρ_max_ = 0.51 e Å^−3^
Δρ_min_ = −0.59 e Å^−3^

### 

Data collection: *APEX2* (Bruker, 2005 ▶); cell refinement: *SAINT* (Bruker, 2005 ▶); data reduction: *SAINT*; program(s) used to solve structure: *SHELXTL* (Sheldrick, 2008 ▶); program(s) used to refine structure: *SHELXTL*; molecular graphics: *SHELXTL*; software used to prepare material for publication: *PLATON* (Spek, 2009 ▶).

## Supplementary Material

Crystal structure: contains datablocks global, I. DOI: 10.1107/S1600536811017776/jh2288sup1.cif

Structure factors: contains datablocks I. DOI: 10.1107/S1600536811017776/jh2288Isup2.hkl

Additional supplementary materials:  crystallographic information; 3D view; checkCIF report

## Figures and Tables

**Table:** 

Ag1—N1	2.141 (2)
Ag1—N3	2.147 (2)
Ag1—O1^i^	2.768 (2)

**Table:** 

N1—Ag1—N3	167.23 (9)
N2—N1—C1	105.1 (2)
N2—N1—Ag1	115.79 (17)
C1—N1—Ag1	137.10 (19)
Ag1^i^—O1—N5	141.8 (2)

Symmetry code: (i) 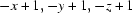.

**Table 2:** Hydrogen-bond geometry (Å, °) *Cg*1 and *Cg*2 are the centroids of the N3/N4/C17–C19 and C5–C10 rings, respectively.

*D*—H⋯*A*	*D*—H	H⋯*A*	*D*⋯*A*	*D*—H⋯*A*
N2—H2⋯O1^i^	0.88	1.97	2.686 (3)	137
N4—H4⋯O3^i^	0.88	1.98	2.858 (3)	175
C26—H26⋯O3^ii^	0.95	2.58	3.358 (4)	140
C32—H32⋯O3^i^	0.95	2.50	3.145 (4)	125
C12—H12⋯*Cg*2^iii^	0.95	2.99	3.437 (4)	111
C30—H30⋯*Cg*3^iv^	0.95	2.98	3.456 (3)	112

Symmetry codes: (i) 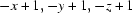; (ii) 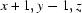; (iii) 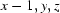; (iv) 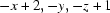.

**Fig. 1.**
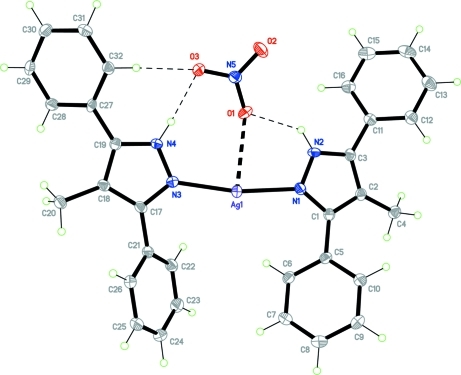
The ORTEP plot of the title compound, showing 50% probability displacement ellipsoids and the atomic numbering. The dashed lines show the hydrogen bondings.

**Fig. 2.**
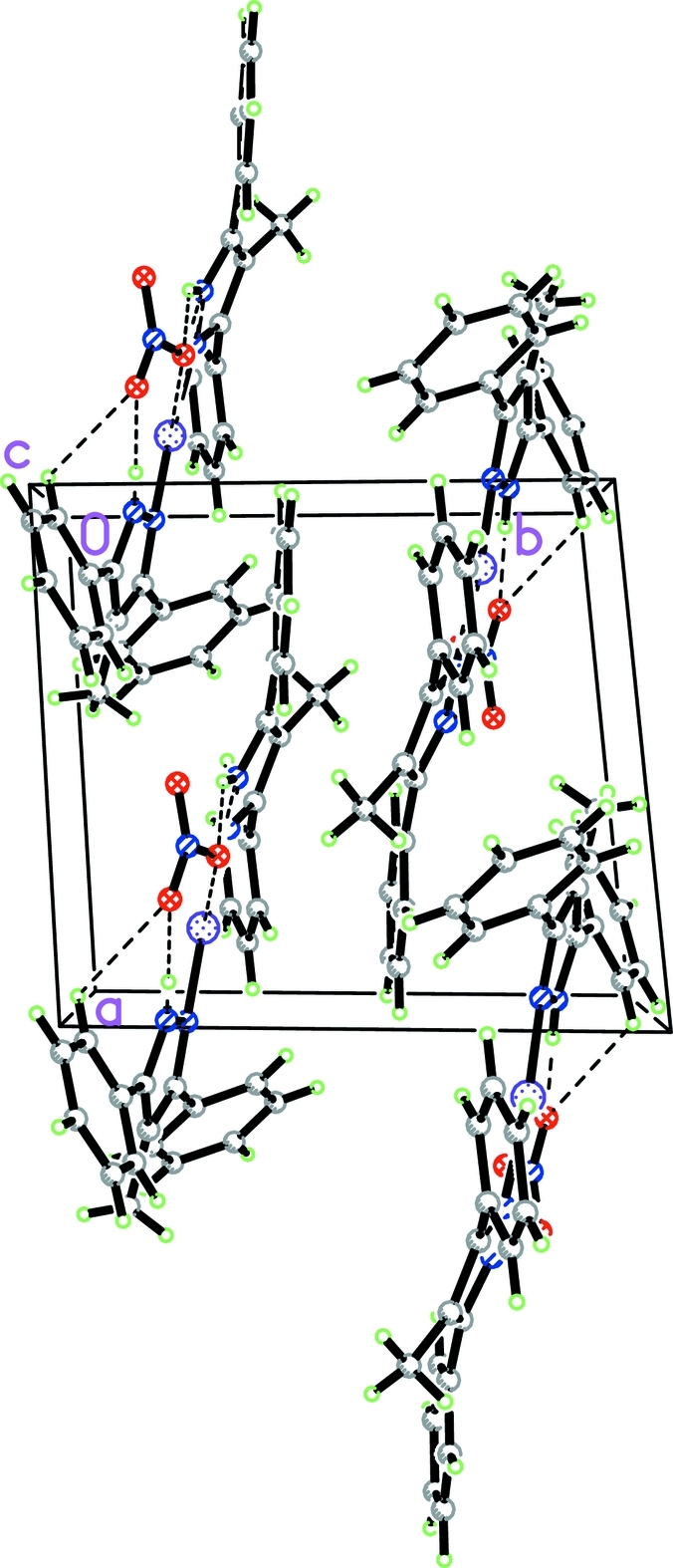
The packing diagram of the title compound viewed down the c-axis. The dashed lines show the intermolecular interactions.

## supplementary crystallographic information

### Comment

The chemistry of coinage metals with pyrazole derived ligands has attracted much interest, mainly to their diverse structures and applications in areas such as modeling C—H bond activations. The abundance and variety of pyrazole complexes of copper is in contrast to the small number of the corresponding silver and gold complexes (Rasika Dias *et al.*, 2007). In continuation of our research on pyrazolate complexes (Hossaini Sadr *et al.*, 2008*a*; Hossaini Sadr *et al.*, 2008*b*; Hossaini Sadr *et al.*, 2006; Hossaini Sadr *et al.*, 2004), we synthesized the title compound and determined its structure by X-ray diffraction.

The asymmetric unit of the title complex, Fig. 1, comprises a cation complex and a nitrate ion. The bond lengths (Allen, *et al.*, 1987) and angles are within the normal ranges. The geometry around Ag(I) is T-shaped which is coordinated by two pyrazolate ligands and a nitrate ion. The crystal structure is stabilized by the intermolecular N—H···O, C—H···O and C—H···π interactions (Table 1).

### Experimental

To an acetone (40 ml) solution of 4-methyl-3,5-diphenyl-1*H*-pyrazole (0.1 g, 1 mmol) under a dry nitrogen atmosphere, AgNO_3_ (0.07 g, 1 mmol) was added and the solution was stirred for 3 h. The resulting mixture was filtered and the precipitate was washed with cold acetone (2 *X* 10 ml). The bright yellow precipitate was dissolved in acetonitrile and the filtrate was left to evaporate slowly at ambient temperature. Single crystals suitable for X-ray diffraction analysis were obtained after 4 days.

### Refinement

All hydrogen atoms were positioned geometrically with C–H = 0.95–0.98 Å and included in a riding model approximation with *U*_iso_ (H) = 1.2 or 1.5 *U*_eq_ (C), except the N-bound H atoms which was located from the difference Fourier map and constrained to refine with the parent atom with *U*_iso_ (H) = 1.2 *U*_eq_ (N). A rotating model were applied to the methyl groups.

### Crystal data

**Table:**

[Ag(C_16_H_14_N_2_)_2_]NO_3_	*Z* = 2
*M**_r_* = 638.46	*F*(000) = 652
Triclinic, *P*1	*D*_x_ = 1.569 Mg m^−^^3^
Hall symbol: -P 1	Mo *K*α radiation, λ = 0.71073 Å
*a* = 10.5529 (12) Å	Cell parameters from 5736 reflections
*b* = 10.8791 (13) Å	θ = 2.4–28.2°
*c* = 12.8396 (15) Å	µ = 0.79 mm^−^^1^
α = 80.454 (2)°	*T* = 100 K
β = 68.806 (2)°	Plate, colorless
γ = 82.398 (2)°	0.45 × 0.25 × 0.08 mm
*V* = 1351.1 (3) Å^3^	

### Data collection

**Table:**

Bruker APEXII CCD area-detector diffractometer	4588 independent reflections
Radiation source: fine-focus sealed tube	4127 reflections with *I* > 2σ(*I*)
graphite	*R*_int_ = 0.022
φ and ω scans	θ_max_ = 25.0°, θ_min_ = 1.7°
Absorption correction: multi-scan (*SADABS*; Bruker, 2005)	*h* = −12→12
*T*_min_ = 0.717, *T*_max_ = 0.940	*k* = −12→12
8897 measured reflections	*l* = −15→15

### Refinement

**Table:**

Refinement on *F*^2^	Primary atom site location: structure-invariant direct methods
Least-squares matrix: full	Secondary atom site location: difference Fourier map
*R*[*F*^2^ > 2σ(*F*^2^)] = 0.029	Hydrogen site location: inferred from neighbouring sites
*wR*(*F*^2^) = 0.084	H-atom parameters constrained
*S* = 1.15	*w* = 1/[σ^2^(*F*_o_^2^) + (0.0396*P*)^2^ + 1.0248*P*] where *P* = (*F*_o_^2^ + 2*F*_c_^2^)/3
4588 reflections	(Δ/σ)_max_ < 0.001
372 parameters	Δρ_max_ = 0.51 e Å^−^^3^
0 restraints	Δρ_min_ = −0.59 e Å^−^^3^

### Special details

**Table:**

Experimental. Ratio of minimum to maximum apparent transmission: 0.450769
Geometry. All e.s.d.'s (except the e.s.d. in the dihedral angle between two l.s. planes) are estimated using the full covariance matrix. The cell e.s.d.'s are taken into account individually in the estimation of e.s.d.'s in distances, angles and torsion angles; correlations between e.s.d.'s in cell parameters are only used when they are defined by crystal symmetry. An approximate (isotropic) treatment of cell e.s.d.'s is used for estimating e.s.d.'s involving l.s. planes.
Refinement. Refinement of *F*^2^ against ALL reflections. The weighted *R*-factor *wR* and goodness of fit *S* are based on *F*^2^, conventional *R*-factors *R* are based on *F*, with *F* set to zero for negative *F*^2^. The threshold expression of *F*^2^ > σ(*F*^2^) is used only for calculating *R*-factors(gt) *etc*. and is not relevant to the choice of reflections for refinement. *R*-factors based on *F*^2^ are statistically about twice as large as those based on *F*, and *R*- factors based on ALL data will be even larger.

### Fractional atomic coordinates and isotropic or equivalent isotropic displacement parameters (Å^2^)

**Table:**

	*x*	*y*	*z*	*U*_iso_*/*U*_eq_	
Ag1	0.85303 (2)	0.22645 (2)	0.355819 (18)	0.02035 (9)	
N1	0.6565 (2)	0.2852 (2)	0.34356 (19)	0.0160 (5)	
N2	0.5557 (2)	0.3086 (2)	0.44095 (19)	0.0190 (5)	
H2	0.5622	0.2860	0.5081	0.023*	
N3	1.0319 (2)	0.1865 (2)	0.40203 (19)	0.0171 (5)	
N4	1.0218 (2)	0.1566 (2)	0.51133 (19)	0.0177 (5)	
H4	0.9456	0.1663	0.5687	0.021*	
C1	0.6057 (3)	0.3341 (3)	0.2613 (2)	0.0177 (6)	
C2	0.4725 (3)	0.3904 (3)	0.3072 (2)	0.0172 (6)	
C3	0.4438 (3)	0.3711 (3)	0.4234 (2)	0.0184 (6)	
C4	0.3885 (3)	0.4665 (3)	0.2438 (2)	0.0207 (6)	
H4A	0.3303	0.4122	0.2305	0.031*	
H4B	0.4487	0.5054	0.1714	0.031*	
H4C	0.3314	0.5317	0.2879	0.031*	
C5	0.6853 (3)	0.3163 (3)	0.1425 (2)	0.0183 (6)	
C6	0.8266 (3)	0.3216 (3)	0.0980 (2)	0.0190 (6)	
H6	0.8733	0.3430	0.1426	0.023*	
C7	0.8994 (3)	0.2956 (3)	−0.0113 (2)	0.0232 (7)	
H7	0.9957	0.2982	−0.0405	0.028*	
C8	0.8325 (3)	0.2659 (3)	−0.0782 (2)	0.0238 (7)	
H8	0.8828	0.2480	−0.1527	0.029*	
C9	0.6910 (3)	0.2624 (3)	−0.0350 (2)	0.0235 (7)	
H9	0.6445	0.2431	−0.0805	0.028*	
C10	0.6181 (3)	0.2871 (3)	0.0747 (2)	0.0218 (6)	
H10	0.5219	0.2842	0.1039	0.026*	
C11	0.3216 (3)	0.3995 (3)	0.5199 (2)	0.0175 (6)	
C12	0.1916 (3)	0.4080 (3)	0.5121 (3)	0.0221 (6)	
H12	0.1822	0.4013	0.4425	0.026*	
C13	0.0766 (3)	0.4261 (3)	0.6060 (3)	0.0273 (7)	
H13	−0.0114	0.4312	0.6005	0.033*	
C14	0.0892 (3)	0.4367 (3)	0.7078 (3)	0.0269 (7)	
H14	0.0100	0.4497	0.7716	0.032*	
C15	0.2172 (3)	0.4285 (3)	0.7166 (3)	0.0271 (7)	
H15	0.2256	0.4350	0.7867	0.033*	
C16	0.3326 (3)	0.4110 (3)	0.6234 (2)	0.0208 (6)	
H16	0.4202	0.4066	0.6296	0.025*	
C17	1.1630 (3)	0.1559 (3)	0.3418 (2)	0.0171 (6)	
C18	1.2370 (3)	0.1068 (3)	0.4130 (2)	0.0170 (6)	
C19	1.1421 (3)	0.1098 (3)	0.5220 (2)	0.0169 (6)	
C20	1.3853 (3)	0.0610 (3)	0.3798 (2)	0.0212 (6)	
H20A	1.4195	0.0447	0.3011	0.032*	
H20B	1.3959	−0.0164	0.4283	0.032*	
H20C	1.4372	0.1247	0.3883	0.032*	
C21	1.2098 (3)	0.1833 (3)	0.2165 (2)	0.0178 (6)	
C22	1.1694 (3)	0.2986 (3)	0.1667 (2)	0.0207 (6)	
H22	1.1097	0.3571	0.2129	0.025*	
C23	1.2154 (3)	0.3282 (3)	0.0512 (3)	0.0253 (7)	
H23	1.1878	0.4073	0.0187	0.030*	
C24	1.3016 (3)	0.2440 (3)	−0.0180 (3)	0.0246 (7)	
H24	1.3342	0.2653	−0.0975	0.029*	
C25	1.3394 (3)	0.1284 (3)	0.0303 (3)	0.0237 (7)	
H25	1.3968	0.0694	−0.0167	0.028*	
C26	1.2947 (3)	0.0973 (3)	0.1465 (2)	0.0194 (6)	
H26	1.3218	0.0176	0.1785	0.023*	
C27	1.1550 (3)	0.0741 (2)	0.6337 (2)	0.0153 (6)	
C28	1.2747 (3)	0.0907 (3)	0.6517 (2)	0.0200 (6)	
H28	1.3497	0.1240	0.5907	0.024*	
C29	1.2845 (3)	0.0590 (3)	0.7577 (2)	0.0199 (6)	
H29	1.3651	0.0728	0.7694	0.024*	
C30	1.1771 (3)	0.0073 (3)	0.8471 (2)	0.0219 (6)	
H30	1.1849	−0.0161	0.9193	0.026*	
C31	1.0588 (3)	−0.0101 (3)	0.8301 (2)	0.0213 (6)	
H31	0.9853	−0.0457	0.8909	0.026*	
C32	1.0469 (3)	0.0244 (3)	0.7246 (2)	0.0175 (6)	
H32	0.9644	0.0140	0.7144	0.021*	
O1	0.2945 (2)	0.7310 (3)	0.42450 (18)	0.0354 (6)	
O2	0.4353 (2)	0.7879 (2)	0.25698 (18)	0.0285 (5)	
O3	0.2167 (2)	0.8156 (2)	0.29417 (17)	0.0279 (5)	
N5	0.3164 (2)	0.7795 (2)	0.32412 (19)	0.0190 (5)	

### Atomic displacement parameters (Å^2^)

**Table:**

	*U*^11^	*U*^22^	*U*^33^	*U*^12^	*U*^13^	*U*^23^
Ag1	0.01459 (13)	0.02873 (15)	0.01819 (13)	−0.00052 (9)	−0.00718 (9)	−0.00177 (9)
N1	0.0128 (11)	0.0170 (12)	0.0174 (12)	0.0011 (10)	−0.0053 (10)	−0.0021 (9)
N2	0.0179 (12)	0.0272 (14)	0.0109 (12)	−0.0039 (11)	−0.0030 (10)	−0.0029 (10)
N3	0.0176 (12)	0.0201 (13)	0.0143 (12)	0.0003 (10)	−0.0068 (10)	−0.0023 (9)
N4	0.0139 (12)	0.0259 (14)	0.0124 (12)	−0.0017 (10)	−0.0028 (10)	−0.0037 (10)
C1	0.0170 (14)	0.0193 (15)	0.0181 (15)	−0.0068 (12)	−0.0055 (12)	−0.0032 (11)
C2	0.0171 (14)	0.0169 (14)	0.0193 (15)	−0.0039 (12)	−0.0073 (12)	−0.0029 (11)
C3	0.0180 (14)	0.0185 (15)	0.0204 (15)	−0.0068 (12)	−0.0061 (12)	−0.0042 (11)
C4	0.0213 (15)	0.0201 (15)	0.0214 (15)	0.0018 (13)	−0.0092 (13)	−0.0035 (12)
C5	0.0218 (15)	0.0171 (15)	0.0169 (14)	−0.0026 (12)	−0.0084 (12)	−0.0003 (11)
C6	0.0205 (15)	0.0197 (15)	0.0182 (15)	−0.0021 (12)	−0.0089 (12)	−0.0009 (11)
C7	0.0196 (15)	0.0262 (17)	0.0209 (15)	−0.0016 (13)	−0.0039 (13)	−0.0025 (12)
C8	0.0292 (17)	0.0239 (16)	0.0160 (15)	−0.0013 (14)	−0.0052 (13)	−0.0031 (12)
C9	0.0291 (17)	0.0260 (17)	0.0200 (16)	−0.0030 (14)	−0.0138 (14)	−0.0027 (12)
C10	0.0190 (15)	0.0256 (16)	0.0203 (15)	−0.0036 (13)	−0.0051 (13)	−0.0042 (12)
C11	0.0174 (14)	0.0127 (14)	0.0199 (15)	−0.0011 (11)	−0.0043 (12)	−0.0008 (11)
C12	0.0214 (16)	0.0186 (15)	0.0250 (16)	0.0014 (13)	−0.0078 (13)	−0.0024 (12)
C13	0.0191 (16)	0.0245 (17)	0.0317 (18)	0.0034 (13)	−0.0044 (14)	−0.0003 (13)
C14	0.0275 (17)	0.0178 (15)	0.0234 (16)	0.0069 (13)	0.0017 (14)	−0.0006 (12)
C15	0.0389 (19)	0.0198 (16)	0.0204 (16)	−0.0034 (14)	−0.0069 (14)	−0.0032 (12)
C16	0.0234 (16)	0.0168 (15)	0.0224 (15)	−0.0029 (13)	−0.0076 (13)	−0.0022 (12)
C17	0.0136 (14)	0.0215 (15)	0.0162 (14)	−0.0051 (12)	−0.0020 (12)	−0.0062 (11)
C18	0.0144 (14)	0.0210 (15)	0.0157 (14)	−0.0043 (12)	−0.0031 (12)	−0.0051 (11)
C19	0.0176 (14)	0.0172 (14)	0.0183 (14)	−0.0035 (12)	−0.0079 (12)	−0.0028 (11)
C20	0.0165 (14)	0.0287 (17)	0.0186 (15)	−0.0010 (13)	−0.0047 (12)	−0.0074 (12)
C21	0.0128 (13)	0.0242 (16)	0.0180 (14)	−0.0054 (12)	−0.0055 (12)	−0.0036 (12)
C22	0.0179 (14)	0.0215 (15)	0.0229 (15)	0.0003 (12)	−0.0064 (13)	−0.0066 (12)
C23	0.0235 (16)	0.0284 (17)	0.0247 (16)	−0.0013 (14)	−0.0110 (14)	0.0001 (13)
C24	0.0215 (15)	0.0346 (18)	0.0164 (15)	−0.0007 (14)	−0.0070 (13)	0.0001 (13)
C25	0.0181 (15)	0.0322 (18)	0.0204 (16)	0.0002 (13)	−0.0046 (13)	−0.0090 (13)
C26	0.0158 (14)	0.0228 (16)	0.0204 (15)	−0.0002 (12)	−0.0075 (12)	−0.0026 (12)
C27	0.0171 (14)	0.0110 (13)	0.0191 (14)	0.0062 (11)	−0.0094 (12)	−0.0040 (11)
C28	0.0185 (15)	0.0237 (16)	0.0174 (15)	−0.0062 (12)	−0.0042 (12)	−0.0029 (12)
C29	0.0187 (14)	0.0202 (15)	0.0246 (16)	0.0042 (12)	−0.0125 (13)	−0.0064 (12)
C30	0.0253 (16)	0.0236 (16)	0.0176 (15)	0.0063 (13)	−0.0104 (13)	−0.0049 (12)
C31	0.0197 (15)	0.0225 (16)	0.0174 (15)	−0.0019 (13)	−0.0013 (12)	−0.0030 (12)
C32	0.0154 (14)	0.0180 (15)	0.0195 (15)	0.0012 (12)	−0.0056 (12)	−0.0069 (11)
O1	0.0279 (12)	0.0592 (17)	0.0178 (12)	0.0029 (12)	−0.0119 (10)	0.0025 (11)
O2	0.0163 (11)	0.0369 (13)	0.0280 (12)	−0.0034 (10)	−0.0004 (10)	−0.0078 (10)
O3	0.0189 (11)	0.0466 (14)	0.0178 (11)	0.0030 (10)	−0.0080 (9)	−0.0040 (10)
N5	0.0200 (13)	0.0225 (13)	0.0163 (12)	−0.0038 (11)	−0.0059 (11)	−0.0063 (10)

### Geometric parameters (Å, °)

**Table:**

Ag1—N1	2.141 (2)	C14—H14	0.9500
Ag1—N3	2.147 (2)	C15—C16	1.383 (4)
Ag1—O1^i^	2.768 (2)	C15—H15	0.9500
N1—N2	1.354 (3)	C16—H16	0.9500
N1—C1	1.354 (4)	C17—C18	1.405 (4)
N2—C3	1.356 (4)	C17—C21	1.492 (4)
N2—H2	0.8807	C18—C19	1.399 (4)
N3—C17	1.347 (4)	C18—C20	1.504 (4)
N3—N4	1.355 (3)	C19—C27	1.472 (4)
N4—C19	1.349 (4)	C20—H20A	0.9800
N4—H4	0.8804	C20—H20B	0.9800
C1—C2	1.411 (4)	C20—H20C	0.9800
C1—C5	1.483 (4)	C21—C22	1.397 (4)
C2—C3	1.396 (4)	C21—C26	1.401 (4)
C2—C4	1.495 (4)	C22—C23	1.379 (4)
C3—C11	1.469 (4)	C22—H22	0.9500
C4—H4A	0.9800	C23—C24	1.387 (4)
C4—H4B	0.9800	C23—H23	0.9500
C4—H4C	0.9800	C24—C25	1.383 (5)
C5—C6	1.397 (4)	C24—H24	0.9500
C5—C10	1.398 (4)	C25—C26	1.389 (4)
C6—C7	1.390 (4)	C25—H25	0.9500
C6—H6	0.9500	C26—H26	0.9500
C7—C8	1.389 (4)	C27—C32	1.396 (4)
C7—H7	0.9500	C27—C28	1.403 (4)
C8—C9	1.396 (4)	C28—C29	1.385 (4)
C8—H8	0.9500	C28—H28	0.9500
C9—C10	1.392 (4)	C29—C30	1.390 (4)
C9—H9	0.9500	C29—H29	0.9500
C10—H10	0.9500	C30—C31	1.384 (4)
C11—C12	1.402 (4)	C30—H30	0.9500
C11—C16	1.402 (4)	C31—C32	1.390 (4)
C12—C13	1.386 (4)	C31—H31	0.9500
C12—H12	0.9500	C32—H32	0.9500
C13—C14	1.385 (5)	O1—N5	1.259 (3)
C13—H13	0.9500	O2—N5	1.244 (3)
C14—C15	1.386 (5)	O3—N5	1.242 (3)
			
N1—Ag1—N3	167.23 (9)	C16—C15—H15	120.0
N2—N1—C1	105.1 (2)	C14—C15—H15	120.0
N2—N1—Ag1	115.79 (17)	C15—C16—C11	120.6 (3)
C1—N1—Ag1	137.10 (19)	C15—C16—H16	119.7
Ag1^i^—O1—N5	141.8 (2)	C11—C16—H16	119.7
N1—N2—C3	112.3 (2)	N3—C17—C18	110.9 (2)
N1—N2—H2	123.8	N3—C17—C21	118.8 (3)
C3—N2—H2	123.9	C18—C17—C21	130.2 (3)
C17—N3—N4	105.3 (2)	C19—C18—C17	104.7 (2)
C17—N3—Ag1	131.80 (19)	C19—C18—C20	127.4 (3)
N4—N3—Ag1	121.04 (17)	C17—C18—C20	127.8 (2)
C19—N4—N3	112.1 (2)	N4—C19—C18	106.9 (2)
C19—N4—H4	123.9	N4—C19—C27	121.2 (2)
N3—N4—H4	123.9	C18—C19—C27	132.0 (3)
N1—C1—C2	111.0 (2)	C18—C20—H20A	109.5
N1—C1—C5	120.1 (3)	C18—C20—H20B	109.5
C2—C1—C5	128.8 (3)	H20A—C20—H20B	109.5
C3—C2—C1	104.6 (3)	C18—C20—H20C	109.5
C3—C2—C4	128.0 (3)	H20A—C20—H20C	109.5
C1—C2—C4	127.1 (3)	H20B—C20—H20C	109.5
N2—C3—C2	107.0 (2)	C22—C21—C26	118.6 (3)
N2—C3—C11	119.9 (3)	C22—C21—C17	119.5 (3)
C2—C3—C11	133.0 (3)	C26—C21—C17	122.0 (3)
C2—C4—H4A	109.5	C23—C22—C21	120.6 (3)
C2—C4—H4B	109.5	C23—C22—H22	119.7
H4A—C4—H4B	109.5	C21—C22—H22	119.7
C2—C4—H4C	109.5	C22—C23—C24	120.8 (3)
H4A—C4—H4C	109.5	C22—C23—H23	119.6
H4B—C4—H4C	109.5	C24—C23—H23	119.6
C6—C5—C10	119.0 (3)	C25—C24—C23	119.1 (3)
C6—C5—C1	121.7 (3)	C25—C24—H24	120.5
C10—C5—C1	119.2 (3)	C23—C24—H24	120.5
C7—C6—C5	120.3 (3)	C24—C25—C26	120.9 (3)
C7—C6—H6	119.9	C24—C25—H25	119.6
C5—C6—H6	119.9	C26—C25—H25	119.6
C8—C7—C6	120.6 (3)	C25—C26—C21	120.0 (3)
C8—C7—H7	119.7	C25—C26—H26	120.0
C6—C7—H7	119.7	C21—C26—H26	120.0
C7—C8—C9	119.5 (3)	C32—C27—C28	118.5 (3)
C7—C8—H8	120.2	C32—C27—C19	120.4 (3)
C9—C8—H8	120.2	C28—C27—C19	121.1 (3)
C10—C9—C8	120.0 (3)	C29—C28—C27	120.5 (3)
C10—C9—H9	120.0	C29—C28—H28	119.8
C8—C9—H9	120.0	C27—C28—H28	119.8
C9—C10—C5	120.6 (3)	C28—C29—C30	120.4 (3)
C9—C10—H10	119.7	C28—C29—H29	119.8
C5—C10—H10	119.7	C30—C29—H29	119.8
C12—C11—C16	118.8 (3)	C31—C30—C29	119.5 (3)
C12—C11—C3	120.6 (3)	C31—C30—H30	120.2
C16—C11—C3	120.5 (3)	C29—C30—H30	120.2
C13—C12—C11	120.1 (3)	C30—C31—C32	120.4 (3)
C13—C12—H12	120.0	C30—C31—H31	119.8
C11—C12—H12	120.0	C32—C31—H31	119.8
C14—C13—C12	120.4 (3)	C31—C32—C27	120.7 (3)
C14—C13—H13	119.8	C31—C32—H32	119.7
C12—C13—H13	119.8	C27—C32—H32	119.7
C13—C14—C15	120.1 (3)	O3—N5—O2	121.6 (2)
C13—C14—H14	119.9	O3—N5—O1	118.2 (2)
C15—C14—H14	119.9	O2—N5—O1	120.2 (2)
C16—C15—C14	120.0 (3)		
			
N3—Ag1—N1—N2	−20.6 (5)	C13—C14—C15—C16	0.7 (5)
N3—Ag1—N1—C1	140.4 (4)	C14—C15—C16—C11	−0.9 (4)
C1—N1—N2—C3	−0.5 (3)	C12—C11—C16—C15	0.9 (4)
Ag1—N1—N2—C3	166.22 (18)	C3—C11—C16—C15	−175.2 (3)
N1—Ag1—N3—C17	−161.4 (3)	N4—N3—C17—C18	−0.3 (3)
N1—Ag1—N3—N4	36.5 (5)	Ag1—N3—C17—C18	−164.46 (19)
C17—N3—N4—C19	0.8 (3)	N4—N3—C17—C21	−177.1 (2)
Ag1—N3—N4—C19	167.07 (18)	Ag1—N3—C17—C21	18.7 (4)
N2—N1—C1—C2	0.9 (3)	N3—C17—C18—C19	−0.3 (3)
Ag1—N1—C1—C2	−161.4 (2)	C21—C17—C18—C19	176.0 (3)
N2—N1—C1—C5	−175.0 (2)	N3—C17—C18—C20	179.7 (3)
Ag1—N1—C1—C5	22.7 (4)	C21—C17—C18—C20	−4.0 (5)
N1—C1—C2—C3	−1.0 (3)	N3—N4—C19—C18	−1.0 (3)
C5—C1—C2—C3	174.4 (3)	N3—N4—C19—C27	178.4 (2)
N1—C1—C2—C4	172.6 (3)	C17—C18—C19—N4	0.7 (3)
C5—C1—C2—C4	−11.9 (5)	C20—C18—C19—N4	−179.2 (3)
N1—N2—C3—C2	−0.1 (3)	C17—C18—C19—C27	−178.6 (3)
N1—N2—C3—C11	177.2 (2)	C20—C18—C19—C27	1.5 (5)
C1—C2—C3—N2	0.7 (3)	N3—C17—C21—C22	43.8 (4)
C4—C2—C3—N2	−172.9 (3)	C18—C17—C21—C22	−132.3 (3)
C1—C2—C3—C11	−176.2 (3)	N3—C17—C21—C26	−136.9 (3)
C4—C2—C3—C11	10.2 (5)	C18—C17—C21—C26	47.0 (4)
N1—C1—C5—C6	−38.9 (4)	C26—C21—C22—C23	−1.8 (4)
C2—C1—C5—C6	146.0 (3)	C17—C21—C22—C23	177.5 (3)
N1—C1—C5—C10	137.7 (3)	C21—C22—C23—C24	0.6 (5)
C2—C1—C5—C10	−37.4 (4)	C22—C23—C24—C25	1.0 (5)
C10—C5—C6—C7	−1.3 (4)	C23—C24—C25—C26	−1.4 (5)
C1—C5—C6—C7	175.3 (3)	C24—C25—C26—C21	0.2 (4)
C5—C6—C7—C8	0.9 (5)	C22—C21—C26—C25	1.4 (4)
C6—C7—C8—C9	0.2 (5)	C17—C21—C26—C25	−177.8 (3)
C7—C8—C9—C10	−0.8 (5)	N4—C19—C27—C32	34.1 (4)
C8—C9—C10—C5	0.4 (5)	C18—C19—C27—C32	−146.7 (3)
C6—C5—C10—C9	0.6 (4)	N4—C19—C27—C28	−145.0 (3)
C1—C5—C10—C9	−176.0 (3)	C18—C19—C27—C28	34.2 (5)
N2—C3—C11—C12	−149.9 (3)	C32—C27—C28—C29	−0.4 (4)
C2—C3—C11—C12	26.7 (5)	C19—C27—C28—C29	178.7 (3)
N2—C3—C11—C16	26.1 (4)	C27—C28—C29—C30	1.7 (4)
C2—C3—C11—C16	−157.3 (3)	C28—C29—C30—C31	−1.4 (4)
C16—C11—C12—C13	−0.6 (4)	C29—C30—C31—C32	−0.2 (4)
C3—C11—C12—C13	175.4 (3)	C30—C31—C32—C27	1.6 (4)
C11—C12—C13—C14	0.4 (5)	C28—C27—C32—C31	−1.2 (4)
C12—C13—C14—C15	−0.4 (5)	C19—C27—C32—C31	179.7 (3)

Symmetry codes: (i) −*x*+1, −*y*+1, −*z*+1.

### Hydrogen-bond geometry (Å, °)

**Table:**

Cg1 and Cg2 are the centroids of the N3/N4/C17–C19 and C5–C10 rings, respectively.

**Table:**

*D*—H···*A*	*D*—H	H···*A*	*D*···*A*	*D*—H···*A*
N2—H2···O1^i^	0.88	1.97	2.686 (3)	137
N4—H4···O3^i^	0.88	1.98	2.858 (3)	175
C26—H26···O3^ii^	0.95	2.58	3.358 (4)	140
C32—H32···O3^i^	0.95	2.50	3.145 (4)	125
C12—H12···Cg2^iii^	0.95	2.99	3.437 (4)	111
C30—H30···Cg3^iv^	0.95	2.98	3.456 (3)	112

Symmetry codes: (i) −*x*+1, −*y*+1, −*z*+1; (ii) *x*+1, *y*−1, *z*; (iii) *x*−1, *y*, *z*; (iv) −*x*+2, −*y*, −*z*+1.
